# A systematic review of the role of inflammatory biomarkers in acute, subacute and chronic non-specific low back pain

**DOI:** 10.1186/s12891-020-3154-3

**Published:** 2020-03-03

**Authors:** Patrick Morris, Kareem Ali, Mackenzie Merritt, Joey Pelletier, Luciana G Macedo

**Affiliations:** McMaster University, 1144 Main St. W. Rooom 441, Hamilton, Ontario Canada

**Keywords:** Non-specific low back pain, Inflammatory biomarkers, TNF-α, IL-6, IL-1β, CRP, Systematic review, Central sensitization

## Abstract

**Background:**

Low back pain (LBP) is one of the greatest contributors to disability in the world and there is growing interest on the role of biomarkers in LBP. To purpose of this review was to analyze available evidence on the relationship between inflammatory biomarkers, clinical presentation, and outcomes in patients with acute, subacute and chronic non-specific low back pain (NSLBP).

**Methods:**

A search was performed in Medline, Embase, Cinahl and Amed databases. Studies which measured levels of inflammatory biomarkers in participants with NSLBP were included. Two reviewers independently screened titles and abstracts, full-texts, and extracted data from included studies. Methodological quality was assessed using the Newcastle Ottawa Quality Assessment Scale. Level of evidence was assessed using the modified GRADE approach for prognostic studies.

**Results:**

Seven primary studies were included in this review. All results assessed using the modified GRADE demonstrated low to very low quality evidence given the small number of studies and small sample. Three studies examined C-reactive protein (CRP), one of which found significantly higher CRP levels in an acute NSLBP group than in controls and an association between high pain intensity and elevated CRP. Three studies examined tumor necrosis factor alpha (TNF-α), two of which found elevated TNF-α in chronic NSLBP participants compared to controls. Two studies examined interleukin 6 (IL-6), none of which found a significant difference in IL-6 levels between NSLBP groups and controls. Two studies examined interleukin 1 beta (IL-β), none of which found a significant difference in IL-β levels between NSLBP groups and controls.

**Conclusions:**

This review found evidence of elevated CRP in individuals with acute NSLBP and elevated TNF-Α in individuals with chronic NSLBP. There are a limited number of high-quality studies evaluating similar patient groups and similar biomarkers, which limits the conclusion of this review.

## Background

Low back pain (LBP) is a common global condition that affects the lives of many adults [1, 2]. LBP was ranked in the Global Burden of Diseases by Vos et al. [1, 2], as one of the greatest contributors to global disability. The World Health Organization recognizes low back pain as the leading cause of activity and work limitation throughout much of the world [3]. It is estimated that up to 85% of working people can expect to experience LBP during their lifetime [3]. As a result, LBP causes an enormous economic burden on individuals, families, industries and government [4, 5] The direct and indirect costs of LBP in the United States have been estimated to be approximately $100–200 billion dollars annually [5, 6]. In Canada, medical costs alone are estimated to be between $6–12 billion, without factoring time lost at work and costs to society [6].

LBP is defined as pain, tension, or rigidity that occurs between the 12th rib posteriorly and the gluteal line [7]. Non-specific LBP (NSLBP), as defined by the National Institute for Health and Care Excellence [8], is tension, soreness and/or stiffness of unknown etiology in the lower back region with joint, disc and connective tissue involvement potentially contributing to symptoms. In those with NSLBP, the suffering caused by LBP cannot be attributable to a specific diagnosis. It has been estimated that up to 85% of patients with isolated LBP are not given a precise pathoanatomical diagnosis to explain their pain [9, 10]. There is still the possibility that structural deficits play a role in NSLBP, but current diagnostic tools or knowledge does not allow for the identification of such factors.

Given that the majority of patients with LBP are diagnosed with NSLBP, research has recently been directed towards investigating the role central sensitization and other mechanisms may have as contributing factors to LBP in these individuals [11]. Central sensitization has been defined as “an amplification of neural signaling within the central nervous system (CNS) that elicits pain hypersensitivity” [12]. Central sensitization is commonly associated with chronic pain syndromes and is above and beyond specific diagnostic testing and structural issues, thus potentially playing a significant role in cases of NSLBP [13].

Proinflammatory cytokines present in the CNS and circulation, have been implicated in the processes of central sensitization [14, 15]. These are molecular biomarkers which can be objectively measured to determine the nature and progression of pathological processes, such as inflammation [16]. Systemic inflammation has been shown to be a central factor in LBP and elevated levels of inflammatory biomarkers such as TNF-Α, IL-6 and IL-1B, have been shown to increase inflammatory and neuropathic pain [14, 17]. Thus it is possible that a connection exists between levels of these inflammatory biomarkers identified in tissue, blood, or other fluids and outcomes associated with NSLBP.

The purpose of this study was to systematically review the evidence on the relationship between inflammatory biomarkers and the presence of low back pain, symptoms, clinical presentation and outcomes in patients with acute, subacute and chronic NSLBP. A better understanding of the association of pro-inflammatory biomarkers with NSLBP will support the investigation of pro-inflammatory biomarkers as mediators or moderators of interventions that can lead to the development of better-targeted interventions.

## Methods

A protocol for the review was developed a priori using the Cochrane Handbook [18] although the study was not registered on PROSPERO. Reporting of the review was conducted using the PRISMA guidelines [19].

The level of evidence was rated using the modified GRADE [20] approach for prognostic factors research. This modified GRADE approach allows for the evaluation of features specific to prognostic research and can be expanded to other epidemiological evaluations as well as narrative synthesis. Grading starts with the identification of the phase of investigation. Phase 2 and 3 studies conducted to confirm independent associations between outcomes and prognostic factors, and to understand the underlying process for the prognosis respectively, should be judged as high-quality evidence. Phase 1 studies conducted to generate hypothesis provide weaker evidence. Similar factors used for grading interventions studies are then taken into consideration and quality of evidence is downgraded for: study limitations (high risk of bias), inconsistency (unexplained heterogeneity of results assessed either using statistical heterogeneity statistics for meta-analysis or evaluating point estimates and confidence intervals for narrative reviews), indirectness (study characteristics do not reflect review question), imprecision (small sample and imprecise estimates for meta-analysis and no sample size calculation, less than 10 outcome events for each prognostic variable and imprecision of estimates) and finally, publication bias (downgraded unless predictor has been evaluated on a number of phase 2 and 3 studies). In addition, identification of moderate or large effect sizes and the identification of a exposure-response gradient (large amount of predictor is linked to larger or lower effect sizes) should be considered for upgrading the quality of evidence.

### Data sources and searches

A computerized search was developed and performed in collaboration with an experienced university librarian to identify relevant studies. The search was conducted on Ovid MEDLINE Epub Ahead of Print, In-Process, & Other Non-Indexed Citations, Ovid MEDLINE Daily, Ovid MEDLINE 1946 to July 2019, Ovid EMBASE (1974 to July 2019), CINAHL (1982 to July 2019), and AMED (1985 to July 2019). Key words related to the domains of inflammatory biomarkers and LBP were used for searching with subheadings and word truncations, according to each database (see Additional file 1 for search strategy). The searches were not restricted to any specific language.

### Study selection

Studies were retrieved from relevant database and transferred to the reference management software RefWorks. Duplicates were removed electronically and manually. Titles and abstracts were screened for relevance to the topic independently by two reviewers. Full texts were then obtained and screened by two reviewers independently to determine eligibility using screening tools developed a priori. Any disagreements on titles and abstract or full text screening were resolved by a third reviewer (LGM). When more information was needed prior to determining inclusion the authors were contacted.

### Inclusion and exclusion criteria

Studies were considered eligible for inclusion if they were cross sectional, longitudinal cohort, or case control studies and evaluated the relationship between inflammatory biomarkers with symptoms and outcomes of LBP in humans. Cross sectional studies where data is collected at one time point is often used to evaluate prevalence and associations [21]. Cohort studies include prospective and retrospective longitudinal designs and are best to evaluate incidence, natural history and causation [21] Participants are chosen without knowledge of the outcomes and prospectively followed over time (prospective) or recruited posthoc where data has already been collected (retrospective) [21]. Finally, case-control designs often include the recruitment of participants with or without the outcome of interest and exposure is usually determined retrospectively [21]. This design investigates for the relative importance of a predictor in relation to the presence or absence of an outcome. All studies that were not a cross sectional, longitudinal cohort, or case control study were excluded, such as: randomized control trials, animal trials, and systematic reviews.

Studies were included if they involved adults (> 18 years of age) of either gender with NSLBP of any duration (acute, subacute, chronic). Pain can was classified into acute (less than 6 weeks), subacute (6 to 12 weeks) or chronic (greater than 12 weeks) [22]. Studies were excluded if they involved subjects with specific spinal diagnoses such as: spinal stenosis, degenerative disc disease, disc herniation, back pain associated with serious conditions such as: cauda equina syndrome, fracture, systemic or inflammatory diseases, active infection, post-surgical, scoliosis and cancer. Studies investigating genetic correlations with low back pain or that was specifically evaluating the effectiveness of pharmaceutical intervention on pain or anti-cytokine therapy was also excluded.

### Outcomes

Studies were eligible if patient-oriented outcomes were evaluated such as: pain intensity, disability, function, quality of life, return to work, or recurrence were included. Case control studies comparing the presence of low back pain versus controls (healthy) were also eligible.

### Exposure measures

Any biomarkers in the included studies that are known to be involved in inflammatory processes were considered in this review. This included but was not limited to: tumor necrosis factor alpha (TNF-α), c-reactive protein (CRP), interleukin-6 (IL-6), IL-8, and IL-1. There was no limitation to the extraction (CNS or blood) of method of analysis for inflammatory biomarkers.

### Data extraction

Data was extracted from each study independently by two reviewers using a priori designed extraction forms. Characteristics of each study that met the inclusion criteria were recorded in Table 1. Recorded characteristics included: author, title, year published, study design, number of subjects in the exposed and comparison groups, patient demographics, patient outcomes associated with biomarker concentrations, and included biomarkers. Relevant results were recorded for each study, including concentrations of each measured biomarker in exposure and comparison groups as well as the patient outcome value or LBP condition status associated with the concentrations. This data was recorded in Table 4. Any disagreements on data extraction were resolved by a third reviewer (LGM).

### Assessment of quality

Included studies were evaluated for quality and risk of bias using the Newcastle Ottawa Quality Assessment Scale (NOS) for cohort and case control studies (scored out of 9) [31]. For cross-sectional studies the NOS adapted version by Modesti et al. [32], was used (scored out of 10). The NOS scale consists of selection, comparability and exposure or outcome assessment, which can be used to evaluate observational study designs. Two reviewers assessed each study independently using the NOS and subsequently came to a consensus regarding quality. Any discrepancies were resolved collaboratively amongst the five reviewers**.**

## Results

### Search results and study selection

**Fig. 1:**
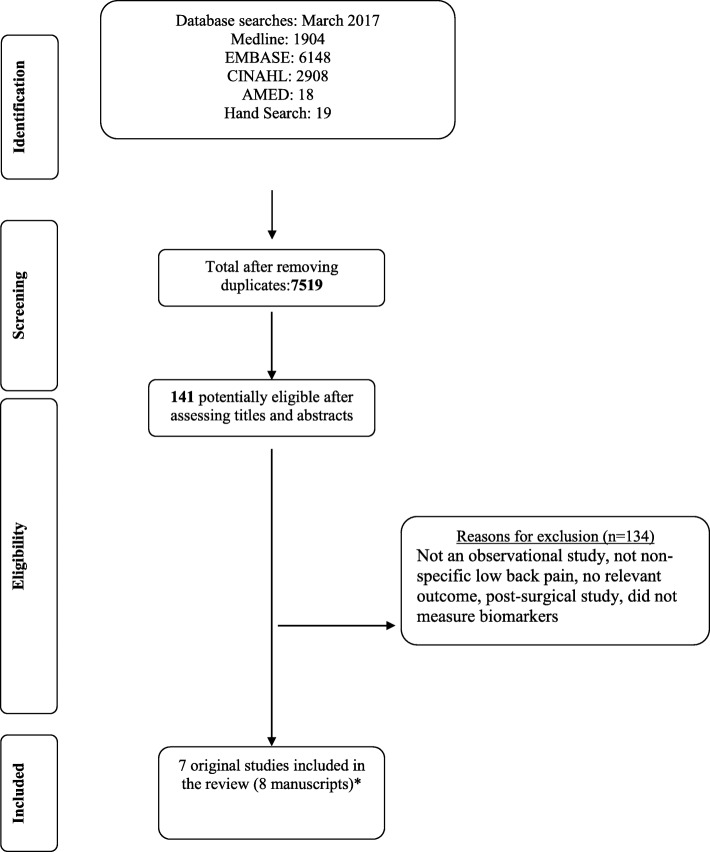
Flow diagram. *Klyne et al.’s study published as case control in 2017 and prospective cohort in 2018

The search conducted in four databases produced 7519 results after removal of duplicates. A total of 141 studies were selected for full-text review based on title and abstract screening. Following full-text screening by two independent reviewers, a total of 133 studies were excluded (Additional file 2 for reasons for exclusion). Studies were most commonly excluded for including participants who had a diagnosis other than NSLBP (e.g. disk herniation, spinal stenosis). A final total of 8 papers reporting on 7 original studies were considered eligible for inclusion in this systematic review (Fig. 1).

### Study characteristics

Seven studies, published between 2005 and 2018, looked at the relationship between inflammatory biomarkers and symptoms, clinical presentation and outcomes in patients with NSLBP. Amongst the seven studies, the total number of participants with NSLBP was 384. Study designs included four case-control [24, 25, 27, 30], three prospective cohort [26, 29, 30], and one cross sectional [29]. Klyne et al’s [25], .case-control study published in 2017 was carried forward into a prospective cohort study in 2018 and reported in a second paper [26] (see Table 1 for full details on the included studies). In Klyne et al’s [25, 26], .study, the following four biomarkers were measured in individuals with acute NSLBP: hsCRP (CRP), IL-6, IL-1β, and TNF-α. The remaining 6 studies examined a single biomarker in chronic NSLBP patients [23, 24, 27–30]. Two studies examined CRP [23, 28], one study examined IL-6 [30], one study examined IL-1β [27], and two studies examined TNF-α [29, 30] (see Table 2 for rationale behind examining each biomarker).

**Table 1:** Study characteristics

Author	Methodological Quality	Study Design	Data Collection	Participant Information
Gebhardt et al., 2006 [23]	6	Prospective cohort NSLBP group received 3 weeks of daily treatment during clinical period (including oral pain medication and physical therapy).	NSLBP group data collected on days 0, 3, 7, 10, 14, 17 and 21 (clinical period) and after 2, 3 and 6 months (follow-up).	41 participants with NSLBP; 1572 controls representative of German population NSLBP defined as over 3 months of chronic myofascial LBP with absence of radicular symptoms or motor deficits.
Heffner et al., 2011 [24]	4	Case-control Chronic NSLBP group compared to healthy control group of age and sex-matched individuals over 24 h period.	Blood samples taken and questionnaires completed in morning of Day 1 (time 1). Sleep quality and pain recorded morning of Day 2 (time 2).	25 participants with chronic NSLBP; 25 age- and sex-matched controls without pain. Chronic NSLBP defined as more than 6 months pain duration without history of inflammatory disease, spine surgery, orthopaedic injury or neurologic signs.
Klyne et al., 2017^a^ [25]	7	Case-control Participants were divided by: (1) those with and without NSLBP, and (2)“high pain,” those with moderate-to-severe NSLBP (VAS ≥4), “low pain,” those with mild LBP (VAS < 4), and controls.	Blood samples collected at one time point and questionnaires completed with 24 h.	99 participants with acute NSLBP; 55 healthy controls Acute NSLBP was defined as an episode within < 2 weeks prior following at least 1 month with no pain. Episodes lasted > 24 h and caused functional limitation and care seeking.
Klyne et al., 2018^a^ [26]	6	Prospective cohort Participants were categorized into NSLBP and control groups. NSLBP participants were then categorized based on their past 6 months of pain and disability status.	Blood samples collected at baseline and 6 months. Pain questionnaires completed within 24 h of sampling and every 2 weeks for 6 months.	Demographics are reported above for Klyne 2017 study. NSLBP participants were categorized in 3 groups based on their NRS pain scores and RMDQ scores at 6 months: (1) unrecovered - increased/unchanged pain and disability from baseline, or a pain score ≥ 7/10 (unrecovered) (2) partially recovered - pain and/or disability is decreased but not yet fully recovered from baseline, and (3) recovered - no pain and disability. NSLBP participants (*N* = 25) without these data were not categorized.
Luchting et al., 2016 [27]	4	Case-control NSLBP group compared to neuropathic pain group and healthy control group	Blood samples taken once between 9:00 and 9:30 AM. Self-reported pain, stress and depression questionnaires administered.	19 participants with chronic NSLBP; 19 participants suffering from neuropathic pain; 19 subjects that are pain-free. NSLBP defined as persistent low back pain not attributable to a detectable pathology
Sturmer et al., 2005 [28]	5	Prospective cohort NSLBP group compared to acute sciatic pain group for duration of 6 months.	Blood samples collected and outcomes assessed on day 3, 7, 10, 14, 17, 21 and after 2, 3, and 6 months.	41 participants with chronic NSLBP (65.8% female, mean age 42.4 years, mean BMI 27.7 kg/m^2^ SD 6.8, 41.5% current smokers) NSLBP defined as LBP for at least 6 weeks attributable to the spine and without signs of specific pathology.
Wang et al., 2010 [29]	7^b^	Prospective Cross-sectional study NSLBP group was compared to a NSLBP with depression group, and healthy age and sex-matched control group at one time point.	Blood samples collected and outcomes assessed at one time point.	29 participants with chronic NSLBP; 29 patients with NSLBP and depression; 29 age and sex matched healthy controls. NSLBP defined as LBP of at least 6 months duration in absence of specific etiology. Leave from work for at least 6 weeks.
Wang et al., 2008 [30]	4	Case-control Matched pair design for a 6 month duration. NSLBP group underwent 3 weeks of inpatient biopsychosocial and physical therapy.	Blood samples collected and outcomes assessed at Day 0, 10, 21 and 6 months.	120 participants with chronic NSLBP; 120 age and sex matched controls with no LBP in the past year (43.3% female, mean age 45.4 SD 11.4 years, mean BMI 27.1 kg/m^2^ (18.7–47.8), 31.7% current smokers) NSLBP defined as unspecific myofascial chronic LBP, present for a least 3 months. Subjects with specific causes of pain or other locations of pain were excluded. Leave from work for at least 6 weeks.

*NOS* Newcastle Ottawa scale, *NSLBP* Non-specific low back pain, *BMI* Body mass index, *LBP* Low back pain, *VAS* Visual analog scale, *SD* Standard deviation, *NRS* Numeric rating scale, *RMDQ* Roland-Morris Disability Questionnaire

^a^Participants in Klyne et al.’s 2018 study taken from the same sample as Klyne et al.’s 2017 study

^b^Newcastle Ottawa Scale (NOS) cross-sectional scale is out of 10

**Table 2:** Proposed significance of biomarkers

Biomarker	Rationale Supporting Examination of Association with NSLBP
hsCRP/CRP	● hsCRP has been found to be associated with patients with osteoarthritis [29] ● It has also been found that individuals with acute sciatic pain also have elevated hsCRP levels [29]
IL-6	● Previous research has shown that proinflammatory cytokines such as IL-6 may be involved in pain processes [31] ● IL-6 has been shown to modulate nociception and possibly contribute to intensifying pain experiences [31] ● Increased IL-6 levels have also been associated with greater pain severity in individuals with rheumatoid arthritis, fibromyalgia and postoperative procedures [31]
TNF-α	● Has been shown to have a role in pathophysiology of discogenic back pain and sciatica [25] ● Has been identified to use in possible treatment strategies of lumbar radicular pain [25] ● Previous studies have shown elevated TNF-α levels in individuals with NSLBP [26]
IL-1β	● Proinflammatory and pro-nociceptive cytokine [24] ● Has been shown to be involved in neurodegeneration, chronic inflammation and chronic pain [24] ● Has been shown to have increased levels in complex regional pain syndrome and chronic tension-type headache [30]

*NSLBP* Non-specific low back pain, *hsCRP* High sensitivity c-reactive protein, *CRP*: c-reactive protein, *IL-6* Interleukin 6, *TNF-*α Tumor necrosis factor alpha, *IL-1β* Interleukin 1 beta

### Methodological quality

The methodological quality of included studies was evaluated using two versions of the Newcastle Ottawa Assessment scale (NOS) [31, 32]. Amongst case-control or cohort studies in this review, the minimum score was 4 out of 9, and maximum score was 7 out of 9, with a median score of 5 (IQR 3). A modified version of the NOS was used for one cross-sectional study, which scored 7 out of 10 [29]. (See Table 3) The most common limitations amongst the studies was a lack of ascertainment of exposure, lack of blinding for outcome assessments and no clear description of controls.

**Table 3:** Newcastle-Ottawa Scale

Author	Year	Selection	Comparability	Outcome	Total Score
Prospective Cohort					
Gebhardt et al. [23],	2006	2/4	1/2	3/3	6/9
Klyne et al. [26],	2018^a^	2/4	2/2	2/3	6/9
Sturmer et al. [28],	2005	2/4	1/2	2/3	5/9
Case-control					
Heffner et al. [24],	2011	1/4	2/2	1/3	4/9
Klyne et al. [25],	2017^a^	4/4	2/2	1/3	7/9
Luchting et al. [27],	2016	2/4	2/2	0/3	4/9
Wang et al. [30],	2008	2/4	1/2	1/3	4/9
Prospective Cross-sectional study					
Wang et al. [29],	2010	4/5	2/2	1/3	7^b^/10

^a^Participants in Klyne et al.’s 2018 study taken from the same sample as Klyne et al.’s 2017 study

^b^Newcastle Ottawa Scale (NOS) cross-sectional scale is out of 10

### Biomarker results

#### C-reactive protein

Three studies compared CRP levels in acute (1 study in 2 publications) [25, 26] and chronic [23, 28] NSLBP patients with healthy controls. One study found a significant difference in CRP levels in the acute NSLBP group compared to healthy controls at baseline [25]. In the same study, significantly higher median CRP levels was also found in those with high pain intensities (VAS ≥ 4), compared to low pain intensities (VAS < 4) and healthy controls [25]. Furthermore, after subdividing acute NSLBP patients into recovered, partially recovered and unrecovered groups at 6 months follow-up, it was found that the recovered group had significantly higher mean CRP levels at baseline than the partially recovered or unrecovered groups [29]. The regression coefficient demonstrated that CRP levels were higher in the recovered group than the unrecovered group by approximately 2.47 μg/mL, demonstrating a difference that is likely clinically important [26]. The remaining two studies did not find a significant difference between CRP levels in chronic NSLBP patients and healthy controls (see Table 4 for detailed results) [29, 30]. Thus, given that only one study is available, there is very low level of evidence from one study that CRP levels is associated with the presence of acute low back pain, levels of acute low back pain and recovery of from acute low back pain with very narrow confidence intervals and therefore a precision on the estimates [29, 30]. These is also low-level evidence, provided there were only two studies with high risk of bias, showing that CRP levels is not associated with the presence or not of chronic low back pain [26, 30].

**Table 4:** Results of studies included in review

Author	Exposure	Results
CRP		
Gebhardt et al., 2006 [23]	NSLBP vs. controls VAS (last 24 h); Functional back capacity score	No significant differences in geometric mean hsCRP levels based on log-transformation between NSLBP and control at baseline (MD = 0.1 95%CI −0.6 to 0.7)). No difference mean change from baseline to 6 months in the NSLBP group (MD = 0.1 95% CI − 0.6 to 0.7).
Sturmer et al., 2005 [28]	NSLBP vs. controls VAS (last 24 h)	There was no difference in hsCRP concentration between pain (> 4.5) compared to low values of pain (≤2.3) (OR = 0.87 (95% CI 0.25 to 3.0)) after adjusting for BMI, age, smoking, alcohol consumption, diabetes and analgesic drugs.
Klyne et al., 2017 [25]	NSLBP vs. controls VAS	CRP was higher in NSLBP participants than controls (*p* = 0.003). Between the three groups, CRP levels were higher in those with high-pain (VAS ≥4) than low-pain **(**VAS < 4) groups (*p* = 0.005) and controls (post-hoc: p = 0.005). Linear and quantile analysis revealed significant positive associations between CRP pain intensity (β = 0.17, 95%CI 0.03 to 0.31)
Klyne et al., 2018 [26]	Recovered, partially recovered, unrecovered VAS RMDQ	CRP levels between the unrecovered group and recovered group (β = 2.47 (95% CI 1.09 to 3.85) and between the partially recovered and recovered group (β = 1.80 (95% CI 0.39 to 3.21) were significant.
IL-6		
Heffner et al., 2011 [24]	NSLBP vs. controls PSQI MPQ-SF	There was no difference in IL-6 levels between NSLBP and control (MD = − 0.1 95%CI − 0.6 to 0.4) IL-6 levels were also associated with higher MPQ-SF affective pain ratings (r = 0.46, *p* = 0.02). Regression analysis showed IL-6 levels were not significantly related to pain (β =1.06; *p* = 0.14).
Klyne et al., 2017 [25]	NSLBP vs. controls VAS	There was no significant difference between NSLBP and controls for IL-6 (*p* = 0.141). Between the three groups IL-6 was higher in high-pain (VAS ≥ 4) than the low-pain group **(**VAS < 4) (*p* = 0.034), but not the control group (*p* = 0.114).
Klyne et al., 2018 [26]	Recovered, partially recovered, unrecovered. VAS RMDQ	No group or session differences were found for IL-6 with results representing narrow confidence intervals.
IL-1		
Luchting et al., 2016 [27]	NSLBP vs. controls	Increased serum levels of IL-1β in patients with neuropathic pain (*p* < 0.05) was found but not in NSLBP (*p* > 0.05).
Klyne et al., 2017 [25]	NSLBP vs. controls VAS	There was no difference in IL-β levels between NSLBP and control (MD = − 0.1 95%CI − 0.6 to 0.4) Between the three groups, there was no difference IL-1β. Linear and quantile analysis revealed significant positive associations between IL-β 80th and 95% quartiles with pain magnification (β = 0.11, 95%CI 0.03 to 0.19) and (β = 0.11, 95%CI 0.03 to 0.18).
Klyne et al., 2018 [26]	Recovered, partially recovered, unrecovered. VAS RMDQ	No group or session differences were found for IL-1β with results representing narrow confidence intervals.
TNF-α		
Wang et al., 2010 [29]	NSLBP vs. controls VAS (last 24 h and past week) RMDQ	Median TNF-α serum levels of patients with chronic NSLBP (2.51 pg/mL) and NSLBP with depression (2.58 pg/mL) were significantly higher than age matched controls (0.1 pg/mL; *p* = 0.004 for NSLBP; *p* = 0.002 for NSLBP + depression). No significant associations were found between TNF-α levels and pain intensity.
Wang et al., 2008 [30]	NSLBP vs. controls VAS RMDQ	Significant difference between groups in percentage of subjects with elevated TNF-α (> 2 pg/mL) at all four time points (baseline OR = 9.5; 95%CI 5.0 to 18.2), (180 days OR = 5.7; 95%CI 3.0 to 11.0). No significant association was found between levels of TNF-α with pain and disability scores.
Klyne et al., 2017 [25]	NSLBP vs. controls VAS	There was no significant difference between NSLBP and controls for TNF-α (*p* = 0.174). There was no difference in TNF-α between the three groups. Linear regression revealed significant an association between TNF-α with pain rumification (β = − 0.20, 95%CI − 0.37 to − 0.02).
Klyne et al., 2018 [26]	Recovered, partially recovered, VAS RMDQ–	TNF-α was lower in the recovered group at both time-points than the other groups. TNF-α was different between the unrecovered vs. recovered groups (β = − 0.68;95% CI − 1.08 to − 0.27) and partially recovery versus recovered (β = − 0.42; 95% CI− 0.72 to − 0.12).
Other		
Luchting et al., 2016 [27]	NSLBP vs. controls	P2RX7 mRNA expression increased in patients with neuropathic pain to controls (MD = -0.6 95%CI − 0.9 to − 0.3), but not in patients NSLBP compared to controls (MD = -0.1 95%CI − 0.4 to 0.2).

*VAS* Visual Analog Scale, *hsCRP* High sensitivity c-reactive protein, *NSLBP* Non-specific low back pain, *MPQ-SF* McGill Pain Questionnaire-Short Form, *IL-6* Interleukin 6, *SD* Standard deviation, *RMDQ* Roland-Morris Disability Questionnaire, *PSQI* Pittsburgh sleep quality index, *CRP* C-reactive protein, *TNF*- ⍺ Tumor necrosis factor alpha, *IL-1β* Interleukin 1 beta, *IQR* Interquartile range, *BMI* Body mass index, *LBP* Low back pain, *NRS* Numeric rating scale, *NPRS* Numeric Pain Rating Scale, *CD4+* Cluster of differentiation 4, *P2RX7* Purinergic Receptor P2X 7, *mRNA* Messenger ribonucleic acid

#### Interleukin-6

Two studies investigating IL-6 compared acute [25, 26] and chronic [24] NSLBP to healthy controls. One study of acute NSLBP patient found no difference in IL-6 levels at baseline between acute NSLBP groups and healthy controls [25]. However, when acute NSLBP participants were divided into a high-pain, low-pain, and control groups, participants with high pain were found to have higher levels of IL-6 than low-pain groups but not controls (see Table 4 for results) [25]. The size of these differences are unknown and therefore it is difficult to make conclusions on clinical significance or whether the findings could have been due to chance given no difference was found for the control group. There was no difference in IL-6 between recovered, unrecovered and partially recovered groups [26]. Heffner et al. [24], found no difference in IL-6 levels between chronic NSLBP groups compared to healthy controls. Given that there was only one study per low back pain duration, there is very low level of evidence from individual studies that serum levels of IL-6 is increased in patients with acute or chronic low back pain versus healthy controls [24, 29]. There is also low level evidence from one study that those with higher levels of acute low back pain have higher concentrations of IL-6 but that IL-6 is not related with recovery status from acute low back pain [24].

#### Interleukin-1β

Two studies investigated the relationship between IL-1β and NSLBP, comparing acute [25, 26] and chronic [27] NSLBP versus healthy controls. Of these studies, none reported any significant increase in levels of IL-1β in acute or chronic NSLBP compared to healthy controls (see Table 4 for results) [24, 25, 29]. Confidence intervals when presented were narrow representing precise estimates. Given that there is only one study per low back pain duration, there is very low level evidence that IL--1β is not increased in patients with either acute or chronic low back pain versus health controls [25–27].)

#### Tumor necrosis factor-⍺

Three studies investigated the relationship in TNF-α between NSLBP and controls, one of the studies investigated acute [25, 26] NSLBP participants and two studies investigated chronic [29, 30] NSLBP patients. One study investigating acute NSLBP found no difference amongst participants with high levels of pain, low levels of pain and controls [25]. However, a significant difference of TNF-α was found when they compared fully recovered NSLBP participants to partially recovered and unrecovered NSLBP participants [26]. The regression coefficient demonstrated a decrease of 0.42 μg/mL in the partially recovered and 0.68 μg/mL in the unrecovered group when compared to the recovered group demonstrating small differences between groups that may not be clinically relevant [26]. Two studies identified a significant difference in TNF-α between NSLBP and controls [29, 30]. One study investigating chronic NSLBP found a significant difference between NSLBP and controls in the percent number of participants with elevated TNF at baseline and all follow up period (day 0, 10, 21, and 180) [27]. Calculated odds ratios were as high 9.5 with a 95% CI of 5.0 to 18.2 demonstrating a large difference in high level concentration of TNF-α in NSLBP. In the same study, no significant difference was found between TNF-α levels and pain (visual analog scale) and function (Roland Morris Disability Questionnaire) [30]. Another study investigating chronic NSLBP found a significant difference in elevated TNF-α values between NSLBP and age matched controls (~ 2.4 μg/mL) [29]. Based on these findings, TNF-α appears to have implications on the presence and recovery of NSLBP however, the degree to which TNF-α impacts specific outcomes of NSLBP has not been identified. Detailed results of each included study are reported in Table 4. There is low level of evidence from one study that TNF-α is not increased in patients with acute low back pain versus healthy controls but that it is associated with recovery from acute low back pain. There is also low-level evidence from two studies of high risk of bias that TNF-α is increased in those with chronic low back pain versus health controls but this increase is not associated with the level of pain or function.

## Discussion

This systematic review examined the association between proinflammatory biomarker concentration levels with acute and chronic NSLBP. Only 3 of the 7 included studies found a significant association between levels of biomarkers and NSLBP and with somewhat questionable clinical important differences/associations [25, 26, 29, 30]. In these 3 studies increased levels of biomarkers, specifically CRP and TNF-α, were associated with the presence of NSLBP [25, 26, 29, 30]. Further breaking down the results of these studies between specific biomarkers, conflicting evidence was found in 2 of 3 studies [23, 28] examining CRP and 1 of 3 studies [25] examining TNF-α. The evidence was very low level to suggest CRP is associated with acute NSLBP and in fact elevated levels may be associated with recovery rather than prolonged NSLBP. A further two studies had low level of evidence to signify any association with chronic NSLBP for CRP. Additionally, TNF-α may be associated with recovery from acute low back pain but the evidence for this is very low. In patients with chronic NSLBP there is low level of evidence of an increase in TNF-α as compared to healthy controls although there is low level evidence that TNF-α is not associated with pain or function. These results suggest that TNF-α may not have a role in those with chronic NSLBP.

No significant associations were found between pro-inflammatory biomarker levels of IL-6 and IL-1β and the presence of chronic NSLBP in any of the included studies that considered these biomarkers, with low to very low levels of evidence. However, participants with acute NSLBP with high pain were found to be associated with higher levels of IL-6 but the evidence was low for this association [24–27].

One previous systematic review has been published examining the relationship between inflammatory biomarkers and NSLBP which found moderate evidence for the relationship between CPR and IL-6 and the NSLBP pain levels, as well as the presence of TNF-α and NSLBP [33]. In contrast to Van den Berg et al. [33], this review has conflicting evidence in finding low to very low evidence of an association between CRP, IL-6 and the degree of NSLBP. In addition, this review found low-level evidence to support the association between TNF-α and NSLBP. The difference in results is likely due to the exclusion of studies that included participants with specific diagnosis of LBP, which may include those with inflammatory conditions that would suggest a stronger association [34–36]. Upon contact of the authors of potentially included studies, it was reported that patients with specific low back pain may have been included [34–36]. Due to the exclusion of these studies this review produces a weaker association between inflammatory biomarkers and NSLBP. Another review by Khan et al. [16], identified positive correlations between pain levels in acute-subacute NSLBP patients with high levels of CRP and IL-6. The conflicting findings between this review and Khan et al. [16], review regarding IL-6 can also be explained again by the decision to exclude three studies [34–36].

One study in this review evaluated patients with acute NSLBP and found that participants with high pain intensity had greater concentrations of CRP than those with low pain intensity and controls, and greater concentration of IL-6 than those with low pain intensity but not controls [25]. It is unclear why those with high levels of pain did not have greater concentration of IL-6 than controls. Through closer observation of graphs, the median and interquartile range (IQR) of CRP and IL-6 were very similar between those with low levels of pain and healthy controls [25]. It is possible that a larger sample size may have been able to identify a difference between the low pain group and controls. Studies that evaluated pain in individuals with specific diagnoses found CRP to be significant but any comparisons with the results of these studies should be made with caution [16]. Another interesting finding by Klyne et al. [26] was that CRP was elevated at baseline in participants who recovered. This may be explained by a reduction in inflammatory processes leading to recovery in pain levels. In addition, an opposite relationship was found for TNF-α, as it was elevated in non-recovered participants at baseline and 6 months follow up [26].

Previous research has demonstrated that increased amounts of inflammation are associated with the development of central sensitization [12, 13]. Higher levels of circulating pro-inflammatory biomarkers, such as CRP and IL-6, have been associated with increased pain in previous studies of patients with specific and NSLBP [16]. However, in this systematic review results are inconclusive regarding the relationship between four inflammatory biomarkers (IL-6, CRP, TNF-α and IL-1β) and NSLBP, therefore, the role these biomarkers have in the development of central sensitization in individuals with NSLBP remains uncertain.

Several limitations have become apparent in this systematic review. The inclusion of multiple study designs and four different biomarkers made it difficult to pool results of the studies. A limited number of studies were eligible for inclusion in general, and few of these studies were comparing the same biomarkers, making it difficult to come to a definitive conclusion on the effects of a particular biomarker on NSLBP. Many studies in this review did not report mean differences and confidence intervals or biomarker concentration levels, which creates uncertainty when determining the clinical importance of the results. A further limitation of the review was not prospectively registering on PROSPERO, however the authors did develop a protocol a priori that was followed throughout. Finally, a lack of clear definitions for the diagnosis of LBP in many studies led to the exclusion of some potentially relevant studies [34–36]. Several strengths can be noted in this review, including the use of PRISMA guidelines for reporting systematic reviews. In addition, an extensive literature search was conducted with the assistance of a health sciences librarian.

Moving forward, there is a greater need for high quality studies to examine a range of inflammatory biomarkers in individuals with NSLBP. This includes high quality longitudinal studies, as well as studies evaluating a wide range of biomarkers and clinical outcomes such as pain, function, and disability. Finally, more longitudinal study designs are needed to examine biomarkers as potential prognostic indicators and correlate concentrations with NSLBP outcomes.

## Conclusions

In conclusion, based on limited evidence, elevated CRP may be found in individuals with acute NSLBP and elevated TNF-α may be found in individuals with chronic NSLBP. Elevated CRP was associated with recovery and elevated TNF-α was associated with lack of recovery in one study of acute NSLBP. The evidence overall was very low to low for all included studies which shows more high-quality studies of biomarker concentrations in individuals with NSLBP are required.

## Supplementary information

**Additional file 1.** Search terms

**Additional file 2.** Excluded studies

## Data Availability

Not applicable.

## Abbreviations

CRP: C-reactive protein
IL: Interleukin
IQR: Interquartile range
NOS: Newcastle Ottawa scale
NSLBP: Non-specific low back pain
TNF-⍺: Tumor necrosis factor alpha
VAS: Visual analogue scale
