# Supplementary material for: A systematic review of the role of inflammatory biomarkers in acute, subacute and chronic non-specific low back pain
Source: BMC Musculoskelet Disord. 2020 Mar 3;21:142. doi: 10.1186/s12891-020-3154-3 (PMC7055034; doi:10.1186/s12891-020-3154-3)
Supplement: Supplementary file 1 — Additional file 1. Search terms [file 12891_2020_3154_MOESM1_ESM.docx]

**Appendix 1**

**Search Terms**

**AMED**

Search Date: July 2019

| 1. exp Low back pain/ |  |
| --- | --- |
| 2. Low back pain.mp. [mp=abstract, heading words, title] |  |
| 3. Back pain.mp. |  |
| 4. exp Backache/ |  |
| 5. backache.mp. |  |
| 6. back-ache.mp. |  |
| 7. lumbago.mp. |  |
| 8. exp Sciatica/ |  |
| 9. sciatica.mp. |  |
| 10. back disorder.mp. |  |
| 11. exp Spinal stenosis/ |  |
| 12. spinal stenosis.mp. |  |
| 13. (low* adj2 back pain).mp. [mp=abstract, heading words, title] |  |
| 14. dorsalgia.mp. |  |
| 15. coccyx.mp. |  |
| 16. discitis.mp. |  |
| 17. exp Intervertebral disk/ |  |
| 18. intervertebral disc.mp. |  |
| 19. spondylosis.mp. |  |
| 20. exp Intervertebral Disk Degeneration/ |  |
| 21. intervertebral disc degeneration.mp. |  |
| 22. degenerative disc disease.mp. |  |
| 23. Intervertebral disc displacement.mp. |  |
| 24. 1 or 2 or 3 or 4 or 5 or 6 or 7 or 8 or 9 or 10 or 11 or 12 or 13 or 14 or 15 or 16 or 17 or 18 or 19 or 20 or 21 or 22 or 23 |  |
| 25. exp biological markers/ |  |
| 26. biological markers.mp. |  |
| 27. biomarkers.mp. |  |
| 28. inflammatory biomarkers.mp. |  |
| 29. exp Cytokines/ |  |
| 30. cytokines.mp. |  |
| 31. exp Interleukins/ |  |
| 32. interleukins*.mp. |  |
| 33. exp Tumor necrosis factor/ |  |
| 34. tumor necrosis factor.mp. |  |
| 35. tumor necrosis factor-alpha.mp. |  |
| 36. c-reactive protein.mp. |  |
| 37. interleukin-6.mp. |  |
| 38. interleukin-1.mp. |  |
| 39. interleukin-8.mp. |  |
| 40. 25 or 26 or 27 or 28 or 29 or 30 or 31 or 32 or 33 or 34 or 35 or 36 or 37 or 38 or 39 |  |
| 41. 24 and 40 |  |

**CINAHL**

Search Date: July 2019

1. "Low Back Pain"
2. "Back Pain+"
3. ["backache"](http://web.b.ebscohost.com.libaccess.lib.mcmaster.ca/ehost/resultsadvanced?vid=74&sid=72d14cfd-4319-494f-b704-22a87d12fb58%40sessionmgr120&bquery=%22biomarkers%22+OR+((MH+%22Biological+Markers%2b%22))+OR+(%22inflammatory+biomarkers%22)+OR+(%22inflammatory+biological+markers%22)+OR+((MH+%22Cytokines%2b%22))+OR+%22cytokines%22+OR+((MH+%22Tumor+Necrosis+Factor%22)+OR+%22tumor+necrosis+factor+alpha%22)+OR+((MH+%22C-Reactive+Protein%22))+OR+%22interleukin-6%22+OR+((MH+%22Interleukin+1%22))+OR+%22interleukin-8%22&bdata=JmRiPWNpbjIwJnR5cGU9MSZzaXRlPWVob3N0LWxpdmU%3d)
4. ["lumbago"](http://web.b.ebscohost.com.libaccess.lib.mcmaster.ca/ehost/resultsadvanced?vid=74&sid=72d14cfd-4319-494f-b704-22a87d12fb58%40sessionmgr120&bquery=%22biomarkers%22+OR+((MH+%22Biological+Markers%2b%22))+OR+(%22inflammatory+biomarkers%22)+OR+(%22inflammatory+biological+markers%22)+OR+((MH+%22Cytokines%2b%22))+OR+%22cytokines%22+OR+((MH+%22Tumor+Necrosis+Factor%22)+OR+%22tumor+necrosis+factor+alpha%22)+OR+((MH+%22C-Reactive+Protein%22))+OR+%22interleukin-6%22+OR+((MH+%22Interleukin+1%22))+OR+%22interleukin-8%22&bdata=JmRiPWNpbjIwJnR5cGU9MSZzaXRlPWVob3N0LWxpdmU%3d)
5. "Sciatica"
6. ["back disorder"](http://web.b.ebscohost.com.libaccess.lib.mcmaster.ca/ehost/resultsadvanced?vid=74&sid=72d14cfd-4319-494f-b704-22a87d12fb58%40sessionmgr120&bquery=%22biomarkers%22+OR+((MH+%22Biological+Markers%2b%22))+OR+(%22inflammatory+biomarkers%22)+OR+(%22inflammatory+biological+markers%22)+OR+((MH+%22Cytokines%2b%22))+OR+%22cytokines%22+OR+((MH+%22Tumor+Necrosis+Factor%22)+OR+%22tumor+necrosis+factor+alpha%22)+OR+((MH+%22C-Reactive+Protein%22))+OR+%22interleukin-6%22+OR+((MH+%22Interleukin+1%22))+OR+%22interleukin-8%22&bdata=JmRiPWNpbjIwJnR5cGU9MSZzaXRlPWVob3N0LWxpdmU%3d)
7. "Spinal Stenosis"
8. ["dorsalgia"](http://web.b.ebscohost.com.libaccess.lib.mcmaster.ca/ehost/resultsadvanced?vid=74&sid=72d14cfd-4319-494f-b704-22a87d12fb58%40sessionmgr120&bquery=%22biomarkers%22+OR+((MH+%22Biological+Markers%2b%22))+OR+(%22inflammatory+biomarkers%22)+OR+(%22inflammatory+biological+markers%22)+OR+((MH+%22Cytokines%2b%22))+OR+%22cytokines%22+OR+((MH+%22Tumor+Necrosis+Factor%22)+OR+%22tumor+necrosis+factor+alpha%22)+OR+((MH+%22C-Reactive+Protein%22))+OR+%22interleukin-6%22+OR+((MH+%22Interleukin+1%22))+OR+%22interleukin-8%22&bdata=JmRiPWNpbjIwJnR5cGU9MSZzaXRlPWVob3N0LWxpdmU%3d)
9. "Coccyx"
10. "Discitis"
11. "intervertebral disc"
12. "Intervertebral Disk+"
13. "Spondylosis+"
14. "Spondylolisthesis"
15. "Spondylolysis+"
16. "Coccydynia"
17. "Intervertebral Disk Displacement"
18. ["intervertebral disc degeneration"](http://web.b.ebscohost.com.libaccess.lib.mcmaster.ca/ehost/resultsadvanced?vid=74&sid=72d14cfd-4319-494f-b704-22a87d12fb58%40sessionmgr120&bquery=%22biomarkers%22+OR+((MH+%22Biological+Markers%2b%22))+OR+(%22inflammatory+biomarkers%22)+OR+(%22inflammatory+biological+markers%22)+OR+((MH+%22Cytokines%2b%22))+OR+%22cytokines%22+OR+((MH+%22Tumor+Necrosis+Factor%22)+OR+%22tumor+necrosis+factor+alpha%22)+OR+((MH+%22C-Reactive+Protein%22))+OR+%22interleukin-6%22+OR+((MH+%22Interleukin+1%22))+OR+%22interleukin-8%22&bdata=JmRiPWNpbjIwJnR5cGU9MSZzaXRlPWVob3N0LWxpdmU%3d)
19. ["degenerative disc disease"](http://web.b.ebscohost.com.libaccess.lib.mcmaster.ca/ehost/resultsadvanced?vid=74&sid=72d14cfd-4319-494f-b704-22a87d12fb58%40sessionmgr120&bquery=%22biomarkers%22+OR+((MH+%22Biological+Markers%2b%22))+OR+(%22inflammatory+biomarkers%22)+OR+(%22inflammatory+biological+markers%22)+OR+((MH+%22Cytokines%2b%22))+OR+%22cytokines%22+OR+((MH+%22Tumor+Necrosis+Factor%22)+OR+%22tumor+necrosis+factor+alpha%22)+OR+((MH+%22C-Reactive+Protein%22))+OR+%22interleukin-6%22+OR+((MH+%22Interleukin+1%22))+OR+%22interleukin-8%22&bdata=JmRiPWNpbjIwJnR5cGU9MSZzaXRlPWVob3N0LWxpdmU%3d)
20. [S1 OR S2 OR S3 OR S4 OR S5 OR S6 OR S7 OR S8 OR S9 OR S10 OR S11 OR S12 OR S13 OR S14 OR S15 OR S16 OR S17 OR S18 OR S19](http://web.b.ebscohost.com.libaccess.lib.mcmaster.ca/ehost/resultsadvanced?vid=83&sid=72d14cfd-4319-494f-b704-22a87d12fb58%40sessionmgr120&bquery=%22biomarkers%22+OR+((MH+%22Biological+Markers%2b%22))+OR+(%22inflammatory+biomarkers%22)+OR+(%22inflammatory+biological+markers%22)+OR+((MH+%22Cytokines%2b%22))+OR+%22cytokines%22+OR+((MH+%22Tumor+Necrosis+Factor%22)+OR+%22tumor+necrosis+factor+alpha%22)+OR+((MH+%22C-Reactive+Protein%22))+OR+%22interleukin-6%22+OR+((MH+%22Interleukin+1%22))+OR+%22interleukin-8%22&bdata=JmRiPWNpbjIwJnR5cGU9MSZzaXRlPWVob3N0LWxpdmU%3d)
21. ["biomarkers"](http://web.b.ebscohost.com.libaccess.lib.mcmaster.ca/ehost/resultsadvanced?vid=84&sid=72d14cfd-4319-494f-b704-22a87d12fb58%40sessionmgr120&bquery=%22biomarkers%22+OR+((MH+%22Biological+Markers%2b%22))+OR+(%22inflammatory+biomarkers%22)+OR+(%22inflammatory+biological+markers%22)+OR+((MH+%22Cytokines%2b%22))+OR+%22cytokines%22+OR+((MH+%22Tumor+Necrosis+Factor%22)+OR+%22tumor+necrosis+factor+alpha%22)+OR+((MH+%22C-Reactive+Protein%22))+OR+%22interleukin-6%22+OR+((MH+%22Interleukin+1%22))+OR+%22interleukin-8%22&bdata=JmRiPWNpbjIwJnR5cGU9MSZzaXRlPWVob3N0LWxpdmU%3d)
22. "Biological Markers+"
23. "inflammatory biomarkers"
24. ["inflammatory biological markers"](http://web.b.ebscohost.com.libaccess.lib.mcmaster.ca/ehost/resultsadvanced?vid=84&sid=72d14cfd-4319-494f-b704-22a87d12fb58%40sessionmgr120&bquery=%22biomarkers%22+OR+((MH+%22Biological+Markers%2b%22))+OR+(%22inflammatory+biomarkers%22)+OR+(%22inflammatory+biological+markers%22)+OR+((MH+%22Cytokines%2b%22))+OR+%22cytokines%22+OR+((MH+%22Tumor+Necrosis+Factor%22)+OR+%22tumor+necrosis+factor+alpha%22)+OR+((MH+%22C-Reactive+Protein%22))+OR+%22interleukin-6%22+OR+((MH+%22Interleukin+1%22))+OR+%22interleukin-8%22&bdata=JmRiPWNpbjIwJnR5cGU9MSZzaXRlPWVob3N0LWxpdmU%3d)
25. "Cytokines+"
26. ["cytokines"](http://web.b.ebscohost.com.libaccess.lib.mcmaster.ca/ehost/resultsadvanced?vid=84&sid=72d14cfd-4319-494f-b704-22a87d12fb58%40sessionmgr120&bquery=%22biomarkers%22+OR+((MH+%22Biological+Markers%2b%22))+OR+(%22inflammatory+biomarkers%22)+OR+(%22inflammatory+biological+markers%22)+OR+((MH+%22Cytokines%2b%22))+OR+%22cytokines%22+OR+((MH+%22Tumor+Necrosis+Factor%22)+OR+%22tumor+necrosis+factor+alpha%22)+OR+((MH+%22C-Reactive+Protein%22))+OR+%22interleukin-6%22+OR+((MH+%22Interleukin+1%22))+OR+%22interleukin-8%22&bdata=JmRiPWNpbjIwJnR5cGU9MSZzaXRlPWVob3N0LWxpdmU%3d)
27. "Tumor Necrosis Factor") OR "tumor necrosis factor alpha"
28. "C-Reactive Protein"
29. ["interleukin-6"](http://web.b.ebscohost.com.libaccess.lib.mcmaster.ca/ehost/resultsadvanced?vid=84&sid=72d14cfd-4319-494f-b704-22a87d12fb58%40sessionmgr120&bquery=%22biomarkers%22+OR+((MH+%22Biological+Markers%2b%22))+OR+(%22inflammatory+biomarkers%22)+OR+(%22inflammatory+biological+markers%22)+OR+((MH+%22Cytokines%2b%22))+OR+%22cytokines%22+OR+((MH+%22Tumor+Necrosis+Factor%22)+OR+%22tumor+necrosis+factor+alpha%22)+OR+((MH+%22C-Reactive+Protein%22))+OR+%22interleukin-6%22+OR+((MH+%22Interleukin+1%22))+OR+%22interleukin-8%22&bdata=JmRiPWNpbjIwJnR5cGU9MSZzaXRlPWVob3N0LWxpdmU%3d)
30. "Interleukin 1"
31. ["interleukin-8"](http://web.b.ebscohost.com.libaccess.lib.mcmaster.ca/ehost/resultsadvanced?vid=84&sid=72d14cfd-4319-494f-b704-22a87d12fb58%40sessionmgr120&bquery=%22biomarkers%22+OR+((MH+%22Biological+Markers%2b%22))+OR+(%22inflammatory+biomarkers%22)+OR+(%22inflammatory+biological+markers%22)+OR+((MH+%22Cytokines%2b%22))+OR+%22cytokines%22+OR+((MH+%22Tumor+Necrosis+Factor%22)+OR+%22tumor+necrosis+factor+alpha%22)+OR+((MH+%22C-Reactive+Protein%22))+OR+%22interleukin-6%22+OR+((MH+%22Interleukin+1%22))+OR+%22interleukin-8%22&bdata=JmRiPWNpbjIwJnR5cGU9MSZzaXRlPWVob3N0LWxpdmU%3d)
32. S21 OR S22 OR S23 OR S24 OR S25 OR S26 OR S27 OR S28 OR S29 OR S30 OR S31
33. S20 AND S32

**MEDLINE**

Search Date: July 2019

| 1. exp Low Back Pain/ |  |
| --- | --- |
| 2. low back pain.mp. |  |
| 3. exp Back Pain/ |  |
| 4. backache.mp. |  |
| 5. back-ache.mp. |  |
| 6. lumbago.mp. |  |
| 7. exp SCIATICA/ |  |
| 8. sciatica.mp. |  |
| 9. back disorder.mp. |  |
| 10. exp Spinal Stenosis/ |  |
| 11. spinal stenosis.mp. |  |
| 12. (low* adj2 back pain).mp. [mp=title, abstract, original title, name of substance word, subject heading word, keyword heading word, protocol supplementary concept word, rare disease supplementary concept word, unique identifier, synonyms] |  |
| 13. dorsalgia.mp. |  |
| 14. exp COCCYX/ |  |
| 15. coccyx.mp. |  |
| 16. exp DISCITIS/ |  |
| 17. discitis.mp. |  |
| 18. exp Intervertebral Disc/ |  |
| 19. exp SPONDYLOSIS/ |  |
| 20. spondylosis.mp. [mp=title, abstract, original title, name of substance word, subject heading word, keyword heading word, protocol supplementary concept word, rare disease supplementary concept word, unique identifier, synonyms] |  |
| 21. exp Spondylolisthesis/ |  |
| 22. exp SPONDYLOLYSIS/ |  |
| 23. coccydynia.mp. |  |
| 24. exp Biomarkers/ |  |
| 25. inflammatory biomarkers.mp. [mp=title, abstract, original title, name of substance word, subject heading word, keyword heading word, protocol supplementary concept word, rare disease supplementary concept word, unique identifier, synonyms] |  |
| 26. exp CYTOKINES/ |  |
| 27. cytokines.mp. [mp=title, abstract, original title, name of substance word, subject heading word, keyword heading word, protocol supplementary concept word, rare disease supplementary concept word, unique identifier, synonyms] |  |
| 28. interleukin*.mp. [mp=title, abstract, original title, name of substance word, subject heading word, keyword heading word, protocol supplementary concept word, rare disease supplementary concept word, unique identifier, synonyms] |  |
| 29. exp Tumor Necrosis Factor-alpha/ |  |
| 30. tumor necrosis factor.mp. [mp=title, abstract, original title, name of substance word, subject heading word, keyword heading word, protocol supplementary concept word, rare disease supplementary concept word, unique identifier, synonyms] |  |
| 31. exp C-Reactive Protein/ |  |
| 32. c-reactive protein.mp. [mp=title, abstract, original title, name of substance word, subject heading word, keyword heading word, protocol supplementary concept word, rare disease supplementary concept word, unique identifier, synonyms] |  |
| 33. exp Interleukin-6/ |  |
| 34. exp Interleukin-1/ |  |
| 35. exp Interleukin-8/ |  |
| 36. exp Intervertebral Disc Degeneration/ |  |
| 37. degenerative disc disease.mp. |  |
| 38. exp Intervertebral Disc Displacement/ |  |
| 39. intervertebral dis?.mp. |  |
| 40. 24 or 25 or 26 or 27 or 28 or 29 or 30 or 31 or 32 or 33 or 34 or 35 |  |
| 41. 1 or 2 or 3 or 4 or 5 or 6 or 7 or 8 or 9 or 10 or 11 or 12 or 13 or 14 or 15 or 16 or 17 or 18 or 19 or 20 or 21 or 22 or 23 or 36 or 37 or 38 or 39 |  |
| 42. 40 and 41 |  |

**EMBASE**

Search Date: July 2019

| 1. low back pain.mp. or exp low back pain/ |  |
| --- | --- |
| 2. backache.mp. or exp backache/ |  |
| 3. back pain.mp. |  |
| 4. back-ache.mp. |  |
| 5. sciatica.mp. or exp sciatica/ |  |
| 6. intervertebral disk degeneration/ or intervertebral dis* degeneration.mp. |  |
| 7. back disorder.mp. |  |
| 8. exp vertebral canal stenosis/ |  |
| 9. spinal stenosis.mp. |  |
| 10. (low* adj2 back pain).mp. |  |
| 11. dorsalgia.mp. |  |
| 12. exp coccyx/ |  |
| 13. coccyx.mp. |  |
| 14. exp spondylosis/ or spondylosis.mp. |  |
| 15. exp spondylolisthesis/ |  |
| 16. exp spondylolysis/ |  |
| 17. coccydnia.mp. |  |
| 18. degenerative disc disease.mp. |  |
| 19. intervertebral disc disease.mp. |  |
| 20. intervertebral disc displacement.mp. |  |
| 21. lumbago.mp. |  |
| 22. 1 or 2 or 3 or 4 or 5 or 6 or 7 or 8 or 9 or 10 or 11 or 12 or 13 or 14 or 15 or 16 or 17 or 18 or 19 or 20 or 21 |  |
| 23. biological marker.mp. or exp biological marker/ |  |
| 24. biomarkers.mp. |  |
| 25. inflammatory biomarkers.mp. |  |
| 26. cytokine/ or cytokine*.mp. |  |
| 27. interleukin.mp. |  |
| 28. tumor necrosis factor.mp. or exp tumor necrosis factor/ |  |
| 29. c reactive protein.mp. or exp C reactive protein/ |  |
| 30. interleukin-1.mp. or exp interleukin 1/ |  |
| 31. interleukin-8.mp. or exp interleukin 8/ |  |
| 32. 23 or 24 or 25 or 26 or 27 or 28 or 29 or 30 or 31 |  |
| 33. 22 and 32 |  |
| 34. limit 33 to human |  |
