# Supplementary material for: A systematic review of the role of inflammatory biomarkers in acute, subacute and chronic non-specific low back pain
Source: BMC Musculoskelet Disord. 2020 Mar 3;21:142. doi: 10.1186/s12891-020-3154-3 (PMC7055034; doi:10.1186/s12891-020-3154-3)
Supplement: Supplementary file 2 — Additional file 2. Excluded studies [file 12891_2020_3154_MOESM2_ESM.docx]

**Appendix 2: Excluded Studies**

| **Reference** | **Reasons for exclusion** |
| --- | --- |
| C-reactive protein and obesity measurements as predictors of low back pain. J Orthop Sports Phys Ther 2011 01;41(1):A26-7. | Not an observational study |
| Akyol S., Senel Eraslan B., Etyemez H., Tanriverdi T., Hanci M. Catabolic cytokine expressions in patients with degenerative disc disease. Turkish Neurosurgery 2010 2010;20(4):492-499. | Included participants with specific LBP |
| Alkhatib B., Rosenzweig D.H., Krock E., Roughley P.J., Beckman L., Steffen T., et al. Acute mechanical injury of the human intervertebral disc: link to degeneration and pain. European Cells and Materials 2014 2014;28:98-111. | Included participants with specific LBP |
| Altun I. Cytokine profile in degenerated painful intervertebral disc: variability with respect to duration of symptoms and type of disease. Spine Journal: Official Journal of the North American Spine Society 2016;16(7):857-861. | No pain or function outcome measures |
| Andrade P, Hoogland G, Garcia MA, Steinbusch HW, Daemen MA, Visser-Vandewalle V. Elevated IL-1beta and IL-6 levels in lumbar herniated discs in patients with sciatic pain. European Spine Journal 2013 Apr;22(4):714-720. | Included participants with specific LBP |
| Andrade P, Hoogland G, Teernstra OP, van Aalst J, van Maren E, Daemen MA, et al. Elevated levels of tumor necrosis factor-alpha and TNFR1 in recurrent herniated lumbar discs correlate with chronicity of postoperative sciatic pain. Spine Journal: Official Journal of the North American Spine Society 2016 Feb;16(2):243-251. | Included participants with specific LBP |
| Andrade P, Visser-Vandewalle V, Philippens M, Daemen MA, Steinbusch HWM, Buurman WA, et al. Tumor necrosis factor-alpha levels correlate with postoperative pain severity in lumbar disc hernia patients: opposite clinical effects between tumor necrosis factor receptor 1 and 2. Pain 2011 Nov;152(11):2645-2652. | Included participants with specific LBP |
| Arnbak B, Hendricks O, Horslev-Petersen K, Jurik AG, Pedersen SJ, Ostergaard M, et al. The discriminative value of inflammatory back pain in patients with persistent low back pain. Scand J Rheumatol 2016 Jul;45(4):321-328. | Included participants with systemic inflammatory disease |
| Aydeniz A., Karsligil T., Gursoy S. Associations between low back pain, disability, functional status, and serum interleukin-1 beta level. Turkish Journal of Medical Sciences 2009 August 2009;39(4):613-617. | Included participants with specific LBP |
| Balague F, Nordin M, Schafer D, Sheikhzadeh A, Lenz ME, Thonar EMA. The potential value of blood biomarkers of intervertebral disk metabolism in the follow-up of patients with sciatica. European Spine Journal 2006 May;15(5):627-633. | Included participants with specific LBP |
| Bozsodi A., Lazary A., Varga PP. Cytokine profile in herniated lumbar disc changes with time of sciatica. European Spine Journal.Conference: EUROSPINE 2014.Lyon France.Conference Publication: (var.pagings) 2014 September 2014;23(SUPPL. 5):S535. | Included participants with specific LBP |
| Briggs MS, Givens DL, Schmitt LC, Taylor CA. Relations of C-reactive protein and obesity to the prevalence and the odds of reporting low back pain. Archives of Physical Medicine & Rehabilitation 2013 Apr;94(4):745-752. | Included participants with specific LBP |
| Brisby H, Olmarker K, Larsson K, Nutu M, Rydevik B. Proinflammatory cytokines in cerebrospinal fluid and serum in patients with disc herniation and sciatica. European Spine Journal 2002 Feb;11(1):62-66. | Included participants with specific LBP |
| Brisby H, Olmarker K, Rosengren L, Cederlund CG, Rydevik B. Markers of nerve tissue injury in the cerebrospinal fluid in patients with lumbar disc herniation and sciatica. Spine 1999 Apr 15;24(8):742-746. | Included participants with specific LBP |
| Burke J.G., Watson R.W.G., McCormack D., Dowling F.E., Walsh M.G., Fitzpatrick JM. Intervertebral discs which cause low back pain secrete high levels of proinflammatory mediators. Journal of Bone and Joint Surgery - Series B 2002 2002;84(2):196-201. | Included participants with specific LBP |
| Chee A., Shi P., An H.S., Markova D., Anderson D.G., Zhang Y. Interleukin-8 present in intervertebral disc tissues from patients with discogenic back pain can stimulate substance p release by cultured sensory neurons. Spine Journal.Conference: 27th Annual Meeting of the North American Spine Society, NASS 2012.Dallas, TX United States.Conference Publication: (var.pagings) 2012 September 2012;12(9 SUPPL. 1):136S. | Included participants with specific LBP |
| Cheng L, Fan W, Liu B, Wang X, Nie L. Th17 lymphocyte levels are higher in patients with ruptured than non-ruptured lumbar discs, and are correlated with pain intensity. Injury 2013 Dec;44(12):1805-1810. | Included participants with specific LBP |
| Corenman DS, Gillard DM, Dornan GJ, Strauch EL. Recombinant human bone morphogenetic protein-2-augmented transforaminal lumbar interbody fusion for the treatment of chronic low back pain secondary to the homogeneous diagnosis of discogenic pain syndrome: two-year outcomes. Spine 2013 Sep 15;38(20):E1269-77. | Post-surgical patients |
| Cuellar JM, Golish SR, Reuter MW, Cuellar VG, Angst MS, Carragee EJ, et al. Cytokine evaluation in individuals with low back pain using discographic lavage. Spine Journal: Official Journal of the North American Spine Society 2010 Mar;10(3):212-218. | Included participants with specific LBP |
| Dagistan Y., Cukur S., Dagistan E., Gezici AR. Role of expression of inflammatory mediators in primary and recurrent lumbar disc herniation. Journal of Korean Neurosurgical Society 2017 January 2017;60(1):40-46. | Included participants with specific LBP |
| Dagistan Y, Cukur S, Dagistan E, Gezici AR. Importance of IL-6, MMP-1, IGF-1, and BAX Levels in Lumbar Herniated Disks and Posterior Longitudinal Ligament in Patients with Sciatic Pain. World Neurosurgery 2015 Dec;84(6):1739-1746. | Included participants with specific LBP |
| De Queiroz B.Z., Pereira D.S., Lopes R.A., Felicio D.C., Silva J.P., De Britto Rosa N.M., et al. Association between the plasma levels of mediators of inflammation with pain and disability in the elderly with acute low back pain: Data from the Back Complaints in the Elders (BACE)-Brazil study. Spine 2016 01 Feb 2016;41(3):197-203. | Contacted authors: Included participants with specific LBP |
| Dongfeng R, Hou S, Wu W, Wang H, Shang W, Tang J, et al. The Expression of Tumor Necrosis Factor-[alpha] and CD68 in High-Intensity Zone of Lumbar Intervertebral Disc on Magnetic Resonance Image in the Patients With Low Back Pain. Spine 2011 03/15;36(6):E429-33. | Included participants with specific LBP |
| Elkan P., StenLinder M., Hedlund R., Willers U., Ponzer S., Gerdhem P. Is there a correlation between plasminogen activator inhibitor-1, c-reactive protein and pain in patients with lumbar disc herniation?. European Spine Journal.Conference: EuroSpine Annual Meeting 2013.Liverpool United Kingdom.Conference Publication: (var.pagings) 2013 September 2013;22(5 SUPPL. 1):S716. | Included participants with specific LBP |
| Fan H.T., Jiang W.H., Zhang B., Wang L., Liu SB. Elevated IL-6 levels correlate with the development of intervertebral disc degeneration: A case-control study and meta-analysis. International Journal of Clinical and Experimental Medicine 2016 29 Feb 2016;9(2):1015-1026. | Included participants with specific LBP |
| Frohner P., Leimert M., Petrowski K. Proinflammatory cytokines as a predictor for the postoperative outcome in minimally invasive lumbar spine procedures. European Spine Journal.Conference: 11th German Spine Conference - Annual Meeting of the German Spine Society.Germany 2016 November 2016;25(11):3814. | Included participants with specific LBP |
| Genevay S, Finckh A, Payer M, Mezin F, Tessitore E, Gabay C, et al. Elevated levels of tumor necrosis factor-alpha in periradicular fat tissue in patients with radiculopathy from herniated disc. Spine 2008 9;33(19):2041-2046. | Included participants with specific LBP |
| Ghafouri B., Carlsson A., Holmberg S., Thelin A., Tagesson C. Biomarkers of systemic inflammation in farmers with musculoskeletal disorders; A plasma proteomic study. BMC Musculoskeletal Disorders 2016;17(1) (pagination):Arte Number: 206. ate of Pubaton: 10 May 2016. | Included participants with conditions other than LBP |
| Goode A.P., Marshall S.W., Kraus V.B., Renner J.B., Sturmer T., Carey T.S., et al. Association between serum and urine biomarkers and lumbar spine individual radiographic features: The Johnston County Osteoarthritis Project. Osteoarthritis and Cartilage 2012 November 2012;20(11):1286-1293. | Did not measure inflammatory biomarkers |
| Goode A.P., Nelson A.E., Kraus V.B., Renner J.B., Jordan JM. Biomarkers reflect differences in osteoarthritis phenotypes of the lumbar spine: the Johnston County Osteoarthritis Project. Osteoarthritis and Cartilage 2017 October 2017;25(10):1672-1679. | Did not measure inflammatory biomarkers |
| Gordh T. A possible biomarker of low back pain: 18F-FDeoxyGlucose uptake in PETscan and CT of the spinal cord. Scandinavian Journal of Pain 2017 01 Apr 2017;15:79-80. | Editorial commentary |
| Grad S, Bow C, Karppinen J, Luk KD, Cheung KM, Alini M, et al. Systemic blood plasma CCL5 and CXCL6: Potential biomarkers for human lumbar disc degeneration. European Cells & Materials 2016 Jan 05;31:1-10. | Included participants with specific LBP |
| Guo T.m., Chen Z.n., Wang Zg. Histopathological changes of discogenic low back pain. Journal of Clinical Rehabilitative Tissue Engineering Research 2011 2011;15(39):7366-7370. | Included participants with specific LBP |
| Habtemariam A, Gronblad M, Virri J, Seitsalo S, Karaharju E. A comparative immunohistochemical study of inflammatory cells in acute-stage and chronic-stage disc herniations. Spine 1998 discussion 2166; Oct 15;23(20):2159-2165. | Included participants with specific LBP |
| Haddad JJ. The role of cytokine-related inflammatory biomarkers in medical conditions of physical trauma and injury: Assessment of serum levels of human interleukin-1beta (hIL-1beta) quantified by sandwich enzyme-linked immunosorbent assay (S-ELISA). Current Trends in Immunology 2015 2015;16:27-37. | Included participants with trauma-related conditions |
| Hashem LE, Roffey DM, Alfasi AM, Papineau GD, Wai DC, Phan P, et al. Exploration of the Inter-Relationships Between Obesity, Physical Inactivity, Inflammation, and Low Back Pain. Spine 2018. | Did not assess relevant outcomes or measures of LBP |
| Hasselhorn HM, Theorell T, Vingard E, Musculoskeletal Intervention Center (MUSIC)-Norrtalje Study Group. Endocrine and immunologic parameters indicative of 6-month prognosis after the onset of low back pain or neck/shoulder pain. Spine 2001 Feb 01;26(3):E24-9. | Included participants with specific LBP |
| Hider S., Konstantinou K., Mattey D.L., Hay EM. Are serum biomarker levels associated with sciatica?. Annals of the Rheumatic Diseases.Conference: Annual European Congress of Rheumatology of the European League Against Rheumatism, EULAR 2015.Rome Italy.Conference Publication: (var.pagings) 2015 June 2015;74(SUPPL. 2):629. | Conference proceedings |
| Hu J.M., Xu X.H., Le ML. Lumbar disc degeneration is associated with local transforming growth factor beta1 and inflammatory cytokines. Chinese Journal of Tissue Engineering Research 2015 2015;19(33):5318-5321. | Included participants with specific LBP |
| Huang K, Lin R, Chen W, Lee C, Yan J, Chang M. IL-20 may contribute to the pathogenesis of human intervertebral disc herniation. Spine 2008 Sep 01;33(19):2034-2040. | Included participants with specific LBP |
| Igarashi A, Kikuchi S, Konno S, Olmarker K. Inflammatory cytokines released from the facet joint tissue in degenerative lumbar spinal disorders. Spine 2004 Oct 01;29(19):2091-2095. | Included participants with specific LBP |
| Jayabalan P., Tremont K., Coelho J.P., Huang W., Cortazzo M.H., Vo N.V., et al. The identification of biomarkers that are predictive of response to interventional spinal procedures for axial low back pain: A pilot study. PM and R.Conference: 2013 Annual Assembly of the American Academy of Physical Medicine and Rehabilitation.National Harbor, MD United States.Conference Publication: (var.pagings) 2013 September 2013;5(9 SUPPL. 1):S296-S297. | Non-observational study design |
| Jiang H, Deng Y, Wang T, Ma J, Li P, Tian P, et al. Interleukin-23 may contribute to the pathogenesis of lumbar disc herniation through the IL-23/IL-17 pathway. Journal of Orthopaedic Surgery 2016 Jan 16;11:12. | Included participants with specific LBP |
| Kang JD, Georgescu HI, McIntyre-Larkin L, Stefanovic-Racic M, Evans CH. Herniated cervical intervertebral discs spontaneously produce matrix metalloproteinases, nitric oxide, interleukin-6, and prostaglandin E2. Spine 1995 Nov 15;20(22):2373-2378. | Included participants with neck pain |
| Kang JD, Stefanovic-Racic M, McIntyre LA, Georgescu HI, Evans CH. Toward a biochemical understanding of human intervertebral disc degeneration and herniation. Contributions of nitric oxide, interleukins, prostaglandin E2, and matrix metalloproteinases. Spine 1997 May 15;22(10):1065-1073. | Included participants with specific LBP |
| Kapoor M., Nakamura A., Rampersaud R., Wu B., Sundararajan K., Rossomacha E., et al. Facet cartilage from patients with lumbar spine osteoarthritis exhibit reduced autophagy and enhanced expression of cell death, inflammatory and catabolic mediators. Osteoporosis International.Conference: World Congress on Osteoporosis, Osteoarthritis and Musculoskeletal Diseases, WCO-IOF-ESCEO 2016.Malaga Spain.Conference Publication: (var.pagings) 2016 April 2016;27(1 SUPPL. 1):S119-S120. | Included participants with specific LBP |
| Kepler CK, Markova DZ, Dibra F, Yadla S, Vaccaro AR, Risbud MV, et al. Expression and Relationship of Proinflammatory Chemokine RANTES/CCL5 and Cytokine IL-1[beta] in Painful Human Intervertebral Discs. Spine 2013 05/15;38(11):873-880. | Included participants with specific LBP |
| Keshari K.R., Lotz J.C., Link T.M., Hu S., Majumdar S., Kurhanewicz J. Lactic acid and proteoglycans as metabolic markers for discogenic back pain. Spine 2008 February 2008;33(3):312-317. | Included participants with specific LBP |
| Khalighi A.R., Saadati N., Farid Hossainy R., Tavakkol Afshari J., Kouhestani Sh., Naghibzadeh B. Role of cytokines in patients with arthritis and low back pain brucellosis. Clinical Microbiology and Infection.Conference: 21st ECCMID/27th ICC.Milan Italy.Conference Publication: (var.pagings) 2011 May 2011;17(SUPPL. 4):S673. | Included participants with an infectious disease |
| Kim J., Ali M.H., An H.S., CsSzabo G., Kroin J.S., Im HJ. Increased inflammatory molecules in degenerative human facet joint capsular tissue. Spine 2010 Annual Meeting of the Cervical Spine Research Society, CSRS;Conference:38th. | Included participants with specific LBP |
| Kim J.H., Moon H.J., Kang J. Annulus fibrosus cells interact with neuron-like cells to modulate production of growth factors and cytokines in symptomatic disc degeneration. Spine Journal.Conference: 26th Annual Meeting of the North American Spine Society, NASS 2011.Chicago, IL United States.Conference Publication: (var.pagings) 2011 October 2011;11(10 SUPPL. 1):3S. | In vitro study design |
| Kim J.S., Ali M.H., Wydra F., Li X., Hamilton J.L., An H.S., et al. Characterization of degenerative human facet joints and facet joint capsular tissues. Osteoarthritis and Cartilage 2015 01 Dec 2015;23(12):2242-2251. | Included participants with specific LBP |
| Kovacs F., Arana E., AsenjoGarcia B., EstremeraRodrigo A., AmengualAlemany G.J., SarasibarEzcurra H., et al. Re: Are Modic changes associated with intervertebral disc cytokine profiles?. Spine Journal 2018 February 2018;18(2):377. | Letter to editor |
| Kraychete DC, Sakata RK, Issy AM, Bacellar O, Santos-Jesus R, Carvalho EM. Serum cytokine levels in patients with chronic low back pain due to herniated disc: analytical cross-sectional study. Sao Paulo Med J 2010;128(5):259-262. | Included participants with specific LBP |
| Kriegeskorte V. Chronic low back pain: Role of TNF-alpha is still unclear. Aktuelle Rheumatol 2008 August 2008;33(4):184-186. | Unable to retrieve full text |
| Le Gars L, Borderie D, Kaplan G, Berenbaum F. Systemic inflammatory response with plasma C-reactive protein elevation in disk-related lumbosciatic syndrome. Joint, Bone, Spine: Revue du Rhumatisme 2000;67(5):452-455. | Included participants with specific LBP |
| Le Maitre C.L., Hoyland J.A., Freemont AJ. Catabolic cytokine expression in degenerate and herniated human intervertebral discs: IL-1beta and TNF-alpha expression profile. Arthritis Research and Therapy 2007;9(4) (pagination):Arte Number: R77. ate of Pubaton: 09 Aug 2007. | Included participants with specific LBP |
| Lee S., Moon C.S., Sul D., Lee J., Bae M., Hong Y., et al. Comparison of growth factor and cytokine expression in patients with degenerated disc disease and herniated nucleus pulposus. Clin Biochem 2009 October 2009;42(15):1504-1511. | Included participants with specific LBP |
| Li S.J., Liang H.J., Ke C.Q., He WD. Correlation of lumbar intervertebral disc herniation and nucleus pulposus inflammatory factor to symptomatic pain. Journal of Clinical Rehabilitative Tissue Engineering Research 2008 27 May 2008;12(22):4303-4307. | Included participants with specific LBP |
| Li Y., Liu J., Liu Z.Z., Duan DP. Inflammation in low back pain may be detected from the peripheral blood: Suggestions for biomarker. Biosci Rep 2016;36(4) (pagination):Arte Number: e00361. ate of Pubaton: 01 Aug 2016. | Did not assess relevant outcomes or measures of LBP |
| Likhitpanichkul M., Torre O.M., Gruen J., Walter B.A., Hecht A.C., Iatridis JC. Do mechanical strain and TNF-alpha interact to amplify pro-inflammatory cytokine production in human annulus fibrosus cells?. J Biomech 2016 03 May 2016;49(7):1214-1220. | Did not assess relevant outcomes or measures of LBP |
| Lippi G, Dagostino C, Buonocore R, Aloe R, Bonaguri C, Fanelli G, et al. The serum concentrations of leptin and MCP-1 independently predict low back pain duration. Clinical Chemistry & Laboratory Medicine 2017 Aug 28;55(9):1368-1374. | Included participants with specific LBP |
| Manicki P., Morel J., Combe B., Van Der Heijde D., Lukas C. Factors associated with elevated acute phase reactants in patients with recent inflammatory back pain: Impact of C reactive protein on the phenotype of patients. Data from the prospective multicenter french cohort desir. Ann Rheum Dis 2016 European Congress of Rheumatology of the European League Against Rheumatism, EULAR;Conference:Annua. | Included participants with systemic inflammatory disease |
| McCooey S. W. Chronic subclinical systemic inflammation as measured by c-reactive protein and its relationship to physical therapy. Orthopaedic Physical Therpy Practice 2008;20(3):128-134. | Not an observational study |
| Menzel NN, Bray-Ward P, Landers M. Diverse roles of cytokines in biological and psychological phenomena: inflammatory cytokine linkages to depression and pain in low back disorders. Commun Nurs Res 2008 2008;41:120-120. | Unable to retrieve full text |
| Nagashima H, Morio Y, Yamane K, Nanjo Y, Teshima R. Tumor necrosis factor-alpha, interleukin-1beta, and interleukin-6 in the cerebrospinal fluid of patients with cervical myelopathy and lumbar radiculopathy. European Spine Journal 2009 Dec;18(12):1946-1950. | Included participants with specific LBP |
| Nakamura A., Rampersaud Y.R., Kapoor M. Facet joint cartilage from patients with lumbar spine osteoarthritis exhibit enhanced inflammatory/catabolic activity associated with dysregulated cartilage homeostasis compared to young patients with intervertebral disc herniation. Annals of the Rheumatic Diseases.Conference: Annual European Congress of Rheumatology of the European League Against Rheumatism, EULAR 2015.Rome Italy.Conference Publication: (var.pagings) 2015 June 2015;74(SUPPL. 2):918. | Included participants with specific LBP |
| Nakamura T., Sugimoto K., Mizuta H. Angiopoietin-like protein 2 induces interleukin-6 expression in the mechanism underlying ligamentum flavum hypertrophy in lumbar spinal canal stenosis patients. Journal of Orthopaedic Translation.Conference: 2016 International Combined Meeting of Orthopaedic Research Societies, ICORS 2016.China 2016 October 2016;7:94. | Included participants with specific LBP |
| Nygaard OP, Mellgren SI, Osterud B. The inflammatory properties of contained and noncontained lumbar disc herniation. Spine 1997 Nov 01;22(21):2484-2488. | Included participants with specific LBP |
| Ohtori S, Inoue G, Ito T, Koshi T, Ozawa T, Doya H, et al. Tumor necrosis factor-immunoreactive cells and PGP 9.5-immunoreactive nerve fibers in vertebral endplates of patients with discogenic low back Pain and Modic Type 1 or Type 2 changes on MRI. Spine 2006 04/20;31(9):1026-1031. | Included participants with specific LBP |
| Ohtori S, Suzuki M, Koshi T, Takaso M, Yamashita M, Inoue G, et al. Proinflammatory cytokines in the cerebrospinal fluid of patients with lumbar radiculopathy. European Spine Journal 2011 Jun;20(6):942-946. | Included participants with specific LBP |
| Park CH, Lee SH. Investigation of High-Sensitivity C-reactive Protein and Erythrocyte Sedimentation Rate in Low Back Pain Patients. The Korean journal of pain 2010 Jun;23(2):147-150. | Included participants with specific LBP |
| Park J, Chang H, Kim Y. The pattern of interleukin-12 and T-helper types 1 and 2 cytokine expression in herniated lumbar disc tissue. Spine 2002 Oct 01;27(19):2125-2128. | Included participants with specific LBP |
| Pedersen LM, Schistad E, Jacobsen LM, Roe C, Gjerstad J. Serum levels of the pro-inflammatory interleukins 6 (IL-6) and -8 (IL-8) in patients with lumbar radicular pain due to disc herniation: A 12-month prospective study. Brain, Behavior, & Immunity 2015 May;46:132-136. | Included participants with specific LBP |
| Phillips K.L.E., Chiverton N., Bunning R.D., Haddock G., Cross A.K., Le Maitre CL. Cytokine and chemokine expression profile in human intervertebral disc degeneration. Rheumatology (United Kingdom).Conference: British Society for Rheumatology and British Health Professionals in Rheumatology Annual Meeting 2012, Rheumatology 2012.Glasgow United Kingdom.Conference Publication: (var.pagings) 2012 May 2012;51(SUPPL. 3):147. | Included participants with specific LBP |
| Phillips K.L.E., Chiverton N., Cole A., Michael A., Breakwell L., Haddock G., et al. Chemokines and their receptors in the human intervertebral disc. Journal of Pathology.Conference: 202nd Scientific Meeting of the Pathological Society of Great Britain and Ireland.Sheffield United Kingdom.Conference Publication: (var.pagings) 2012 September 2012;228(SUPPL. 1):S30. | Conference proceedings |
| Phillips K.L.E., Chiverton N., Haddock G., Bunning R., Cross A., Le Maitre C. Regulation of chemokine expression by Interleukin-1 in human intervertebral disk degeneration. Global Spine Journal 2012 Forum for Spine Research;Conference:Wor. | Included participants with specific LBP |
| Phillips KLE, Cullen K, Chiverton N, Michael ALR, Cole AA, Breakwell LM, et al. Potential roles of cytokines and chemokines in human intervertebral disc degeneration: interleukin-1 is a master regulator of catabolic processes. Osteoarthritis & Cartilage 2015 Jul;23(7):1165-1177. | Post-surgical and post-mortem |
| PielerBruha E. Serum levels of the proinflammatory cytokine interleukin-6 vary based on diagnoses in individuals with lumbar intervertebral disc diseases: Kommentar. Journal fur Mineralstoffwechsel 2016 2016;23(1):21. | Included participants with specific LBP |
| Pritchett JW. C-reactive protein levels determine the severity of soft-tissue injuries. American Journal of Orthopedics (Chatham, Nj) 1996 Nov;25(11):759-761. | Unable to retrieve full text |
| Queiroz B, Pereira DS, de BR, Lopes RA, Felício DC, Pereira DG, et al. Functional performance and plasma cytokine levels in elderly women with and without low back pain. J BACK MUSCULOSKELETAL REHABIL 2015 04;28(2):343-349. | Included participants with specific LBP |
| Rannou F, Ouanes W, Boutron I, Lovisi B, Fayad F, Macé Y, et al. High-sensitivity C-reactive protein in chronic low back pain with vertebral end-plate modic signal changes. ARTHRITIS RHEUM (ARTHRITIS CARE RES) 2007 10/15;57(7):1311-1315. | Included participants with specific LBP |
| Richardson SM, Doyle P, Minogue BM, Gnanalingham K, Hoyland JA. Increased expression of matrix metalloproteinase-10, nerve growth factor and substance P in the painful degenerate intervertebral disc. Arthritis Research & Therapy 2009;11(4):R126. | Post-surgical and post-mortem |
| Roberts S., Eyre D., Atley L., Menage J., Eisenstein SM. Matrix metalloproteinase-generated neoepitopes of collagen type II as potential markers in urine and serum of disc prolapse patients. Int J Exp Pathol 2001 2001;82(6):A17-A18. | Included participants with specific LBP |
| Roy RA, Boucher JP, Comtois AS. Inflammatory response following a short-term course of chiropractic treatment in subjects with and without chronic low back pain. J CHIROPRACT MED 2010 2010;9(3):107-114. | Not an observational study |
| Sainoh T., Orita S., Yamauchi K., Sakuma Y., Kubota G., Oikawa Y., et al. Evaluation of the correlation among the expression of inflammatory cytokines, degeneration of the intervertebral disc, and prominent symptoms in degenerative human lumbar intervertebral discs. Global Spine Journal 2014 Forum for Spine Research;Conference:Wor. | Post-surgical patients |
| Sainoh T, Inage K, Orita S, Koda M, Furuya T, Yamauchi K, et al. Correlation among Inflammatory Cytokine Expression Levels, Degree of Disk Degeneration, and Predominant Clinical Symptoms in Patients with Degenerated Intervertebral Discs. Asian Spine Journal 2017 Jun;11(3):472-477. | Included participants with specific LBP |
| Schell E. Theorell T. Hasson D. Arnetz B. Saraste,H. Stress biomarkers' associations to pain in the neck, shoulder and back in healthy media workers: 12-month prospective follow-up. European Spine Journal 2008 Mar;17(3):393-405. | Included participants with neck and shoulder pain |
| Schistad EI, Espeland A, Pedersen LM, Sandvik L, Gjerstad J, Røe C. Association between baseline IL-6 and 1-year recovery in lumbar radicular pain. Eur J Pain 2014 11;18(10):1394-1401. | Included participants with specific LBP |
| Schroeder G., Markova D., Koemer J.D., Millhouse P.W., Rihn J., Hilibrand A.S., et al. Differential cytokine profiles in intervertebral discs associated with and without modic changes. Journal of Orthopaedic Research 2016 34 (Supplement 1) (no pagination);Conference: 2016 Annual Meeting of the Orthopaedic Research Society. United States:ate of Pubaton: 2016. | Conference proceedings |
| Schroeder M., Viezens L., Schaefer C., Friedrichs B., Algenstaedt P., Ruther W., et al. Chemokine profile of disc degeneration with acute or chronic pain Laboratory investigation. Journal of Neurosurgery: Spine 2013 May 2013;18(5):496-503. | Included participants with specific LBP |
| Scuderi G.J., Brusovanik G.V., Golish S.R., DeMeo R., Hyde J., Hallab N., et al. A critical evaluation of discography in patients with lumbar intervertebral disc disease. Spine Journal 2008 July/August 2008;8(4):624-629. | Included participants with specific LBP |
| Scuderi GJ, Brusovanik GV, Anderson DG, Dunham CJ, Vaccaro AR, Demeo RF, et al. Cytokine assay of the epidural space lavage in patients with lumbar intervertebral disk herniation and radiculopathy. Journal of Spinal Disorders & Techniques 2006 Jun;19(4):266-269. | Included participants with specific LBP |
| Segar A., Fairbank J., Kramer H., Turner S., Menage J., Roberts S., et al. Multi-array and mass spectrometric analysis of plasma from patients with spinal disorders: Attempting to identify novel biomarkers. Osteoarthritis and Cartilage.Conference: 2015 Osteoarthritis Research Society International World Congress, OARSI 2015.Seattle, WA United States.Conference Publication: (var.pagings) 2015 April 2015;23(SUPPL. 2):A378. | Included participants with spinal disorders |
| Shamji MF, Setton LA, Jarvis W, So S, Chen J, Jing L, et al. Proinflammatory cytokine expression profile in degenerated and herniated human intervertebral disc tissues. Arthritis Rheum 2010 07;62(7):1974-1982. | Included participants with specific LBP |
| Skouen JS, Larsen JL, Vollset SE. Cerebrospinal fluid proteins as indicators of nerve root compression in patients with sciatica caused by disc herniation. Spine 1993 Jan;18(1):72-79. | Included participants with specific LBP |
| Smite D. Ancane G. Tretjakovis P. Ancans A. Jurka,A. Relationships between cytokines, emotional and physical disturbances, pain syndrome and level of disability in patients with chronic low back pain. International Journal of Rehabilitation Research 2009 Aug;32(Suppl 1):S103. | Not an observational study |
| Sowa G.A., Agarwal V., Bechara B., Boardman J., Kang J.D., Perera S., et al. Serum biomarkers relate to pain and pain related disability in older adults with low back pain. PM and R.Conference: 2012 American Academy of Physical Medicine and Rehabilitation, AAPM&R Annual Assembly.Atlanta, GA United States.Conference Publication: (var.pagings) 2012 October 2012;4(10 SUPPL. 1):S186-S187. | Conference proceedings |
| Sowa GA, Perera S, Bechara B, Agarwal V, Boardman J, Huang W, et al. Associations Between Serum Biomarkers and Pain and Pain-Related Function in Older Adults with Low Back Pain: A Pilot Study. J Am Geriatr Soc 2014 11;62(11):2047-2055. | Included participants with specific LBP |
| Specchia N, Pagnotta A, Toesca A, Greco F. Cytokines and growth factors in the protruded intervertebral disc of the lumbar spine. European Spine Journal 2002 Apr;11(2):145-151. | Included participants with specific LBP |
| Starkweather A, Witek-Janusek L, Mathews HL. Neural-immune interactions: implications for pain management in patients with low-back pain and sciatica. Biol Res Nurs 2005 01;6(3):196-206. | Not an observational study |
| Sugimori K, Kawaguchi Y, Morita M, Kitajima I, Kimura T. High-sensitivity analysis of serum C-reactive protein in young patients with lumbar disc herniation. Journal of Bone & Joint Surgery - British Volume 2003 Nov;85(8):1151-1154. | Included participants with specific LBP |
| Sutovsky J., Kocmalova M., Benco M., Kazimierova I., Pappova L., Frano A., et al. The role of cytokines in degenerative spine disorders. European Pharmaceutical Journal 2017 01 Sep 2017;64(1):26-29. | Included participants with degenerative spinal disorders |
| Sutovsky J, Benco M, Sutovska M, Kocmalova M, Pappova L, Miklusica J, et al. Cytokine and chemokine profile changes in patients with lower segment lumbar degenerative spondylolisthesis. International Journal Of Surgery 2017 Jul;43:163-170. | Included participants with specific LBP |
| Takahashi H. [A mechanism for sciatic pain caused by lumbar disc herniation--involvement of inflammatory cytokines with sciatic pain]. Nippon Seikeigeka Gakkai Zasshi - Journal of the Japanese Orthopaedic Association 1995 Jan;69(1):17-29. | Included participants with specific LBP |
| Takahashi H, Suguro T, Okazima Y, Motegi M, Okada Y, Kakiuchi T. Inflammatory cytokines in the herniated disc of the lumbar spine. Spine 1996 Jan 15;21(2):218-224. | Included participants with specific LBP |
| Talghini S, Vahedi A, Lotfinia I. Discriminating extrusive and bulging disk herniations by using serum hs CRP. Pakistan Journal of Biological Sciences 2013 Nov 01;16(21):1411-1414. | Included participants with specific LBP |
| Theorell T., Hasselhorn H.M., Vingard E., Andersson B. Interleukin 6 and cortisol in acute musculoskeletal disorders: Results from a case-referent study in Sweden. Stress Med 2000 2000;16(1):27-35. | Included participants with conditions other than LBP |
| Thomas S., Colletta R., Phillips K., Chiverton N., Cole A., Michael A.R., et al. Regulation of neurotrophic factor expression in nerve cells and intervertebral disc cells by inflammatory cytokines, implications for pain pathways. Journal of Pathology.Conference: 202nd Scientific Meeting of the Pathological Society of Great Britain and Ireland.Sheffield United Kingdom.Conference Publication: (var.pagings) 2012 September 2012;228(SUPPL. 1):S18. | Included participants with specific LBP |
| Tufan K, Sen O, Cekinmez M, Bolat FA, Alkan O, Sarica FB, et al. Comparison of E-selectin and the other inflammatory markers in lumbar disc herniation: a new promising therapeutical window for radicular pain. Journal of Spinal Disorders & Techniques 2012 Dec;25(8):443-446. | Included participants with specific LBP |
| Uher T, Bob P. Neuropathic pain, depressive symptoms, and C-reactive protein in sciatica patients. Int J Neurosci 2013 Mar;123(3):204-208. | Included participants with specific LBP |
| Uhoda B. Immunobiological aspects of acute discogenic pain of low back pain. Period Biol 2010 March 2010;112(1):1-5. | Included participants with specific LBP |
| Walden T., Filippi C., Khan A.N., Levine M., Winkelstein B.A., Chahine NO. Serum MMP-9 levels Correlate with the Severity of Intervertebral Disc Herniation in Patients. . | Included participants with specific LBP |
| Wang K., Bao J.P., Yang S., Hong X., Liu L., Xie X.H., et al. A cohort study comparing the serum levels of pro- or anti-inflammatory cytokines in patients with lumbar radicular pain and healthy subjects. European Spine Journal 2016 01 May 2016;25(5):1428-1434. | Included participants with specific LBP |
| Wang K, Bao J, Yang S, Hong X, Liu L, Xie X, et al. A cohort study comparing the serum levels of pro- or anti-inflammatory cytokines in patients with lumbar radicular pain and healthy subjects. European Spine Journal 2016 May;25(5):1428-1434. | Included participants with specific LBP |
| Wang Y, Yi X, Li C. Role of thymic stromal lymphopoietin in the pathogenesis of lumbar disc degeneration. Medicine 2017 Jul;96(30):e7516. | Included participants with specific LBP |
| Wang Z, Wang G, Zhu X, Geng D, Yang H. Interleukin-2 is upregulated in patients with a prolapsed lumbar intervertebral disc and modulates cell proliferation, apoptosis and extracellular matrix metabolism of human nucleus pulposus cells. Experimental & Therapeutic Medicine 2015 Dec;10(6):2437-2443. | Included participants with specific LBP |
| Weber KT, Satoh S, Alipui DO, Virojanapa J, Levine M, Sison C, et al. Exploratory study for identifying systemic biomarkers that correlate with pain response in patients with intervertebral disc disorders. Immunol Res 2015 Dec;63(1-3):170-180. | Included participants with specific LBP |
| Weber KT, Alipui DO, Sison CP, Bloom O, Quraishi S, Overby MC, et al. Serum levels of the proinflammatory cytokine interleukin-6 vary based on diagnoses in individuals with lumbar intervertebral disc diseases. Arthritis Research & Therapy 2016 Jan 07;18:3. | Included participants with specific LBP |
| Wei-Chun Lin, Chao HY, Lung-Chang Chien, Morone NE, Glick RM, Albers KM. The Anti-Inflammatory Actions of Auricular Point Acupressure for Chronic Low Back Pain. EVID BASED COMPLEMENT ALTERN MED 2015 01:1-9. | Non-observational study design |
| Xiaogang M, Quanshan H, Liping Z, Kaken H. The expression of cytokine and its significance for the intervertebral disks of Kazakhs. J Clin Lab Anal 2017 Sep;31(5). | Included participants with specific LBP |
| Xu D, Sun Y, Bao G, Liu W, Zhu X, Cui S, et al. MMP-1 overexpression induced by IL-1beta: possible mechanism for inflammation in degenerative lumbar facet joint. Journal of Orthopaedic Science 2013 Nov;18(6):1012-1019. | Included participants with specific LBP |
| Xue H, Yao Y, Wang X, Zhang F, Jiang X, Liu J, et al. Interleukin-21 Is Associated with the Pathogenesis of Lumbar Disc Herniation. Iranian Journal of Allergy Asthma & Immunology 2015 Oct;14(5):509-518. | Included participants with specific LBP |
| Yang J., Kang J., Feng D., Wang S., Yang H. Increased IL-1 and IL-6 expressions are negatively correlated with modified Japanese Orthopedic Association (mJOA) scores of discogenic low back pain. Xi bao yu fen zi mian yi xue za zhi = Chinese journal of cellular and molecular immunology 2016 01 Jan 2016;32(1):88-91. | Text unavailable |
| Zhang Y., An H., Chee A., Markova D., Anderson G. Cytokine profile in intervertebral disc tissues from patients with discogenic axial back pain confirmed by discography. European Journal of Pain.Conference: 6th Congress of the European Federation of IASP Chapters: Pain in Europe 6th, EFIC.Lisbon Portugal.Conference Publication: (var.pagings) 2009 September 2009;13(SUPPL. 1):S124-S125. | Included participants with specific LBP |
| Zhang Y., Meng Y., Zhao W.D., Huang Y.F., Shen B., Wu DS. Relationship between lumbar spinal stenosis and inflammatory factors in the vein serum of lumbar spinal canal. Chinese Journal of Tissue Engineering Research 2014 2014;18(26):4229-4235. | Included participants with specific LBP |
| Zhang W, Nie L, Guo Y, Han L, Wang X, Zhao H, et al. Th17 Cell Frequency and IL-17 Concentration Correlate With Pre- and Postoperative Pain Sensation in Patients With Intervertebral Disk Degeneration. Orthopedics 2014 07;37(7):e685-91. | Post-surgical patients |
| Zhang Y, Chee A, Shi P, Adams SL, Markova DZ, Anderson DG, et al. Intervertebral Disc Cells Produce Interleukins Found in Patients with Back Pain. American Journal of Physical Medicine & Rehabilitation 2016 Jun;95(6):407-415. | Post-mortem |
| Zhang Y, Zhao Y, Li J, Wang S, Liu Y, Nie L, et al. Interleukin-9 Promotes TNF-α and PGE2 Release in Human Degenerated Intervertebral Disc Tissues. Spine 2016 11;41(21):1631-1640. | Not an observational study |
| Zheng Z., Liu H., Yang H., Wang J., Li Z. Comparison of inflammatory cytokines expression in intervertebral disk protrusion with or without low back pain. Global Spine Journal 2012 Forum for Spine Research;Conference:Wor. | Included participants with specific LBP |
| Zille Queiroz B, Sirineu Pereira D, de BR, Antunes Lopes R, Pereira Andrade AG, Carvalho Felício D, et al. Inflammatory Mediators and Pain in the First Year After Acute Episode of Low-Back Pain in Elderly Women: Longitudinal Data from Back Complaints in the Elders--Brazil. Am J Phys Med Rehabil 2017 08;96(8):535-540. | Included participants with specific LBP |

LBP: low back pain
